# The Junction Between nsp1β and nsp2 in the Porcine Reproductive and Respiratory Syndrome Virus Genome Is a New Site for the Insertion and Expression of Foreign Genes

**DOI:** 10.3390/v17050656

**Published:** 2025-04-30

**Authors:** Changguang Xiao, Yafang Lin, Hailong Zhang, Zongjie Li, Ke Liu, Beibei Li, Donghua Shao, Yafeng Qiu, Zhiyong Ma, Jianchao Wei

**Affiliations:** Shanghai Veterinary Research Institute, Chinese Academy of Agricultural Sciences, Shanghai 200241, China; xiaochangguang@outlook.com (C.X.); 13065714060@163.com (Y.L.); zhanghailong1997@163.com (H.Z.); lizongjie@shvri.ac.cn (Z.L.); liuke@shvri.ac.cn (K.L.); lbb@shvri.ac.cn (B.L.); shaodonghua@shvri.ac.cn (D.S.); yafengq@shvri.ac.cn (Y.Q.)

**Keywords:** porcine reproductive and respiratory syndrome virus, viral vector, recombinant virus, nsp1β, nsp2, foreign gene expression

## Abstract

Porcine reproductive and respiratory syndrome virus (PRRSV) is considered a promising viral vector for the expression and delivery of foreign genes for the development of a new generation of multi-valent vaccines against PRRSV and other porcine viruses, as well as for analyses of the immune response against PRRSV and anti-PRRSV component screening. In the present study, the junction site between nsp1β and nsp2 in the PRRSV genome was tested for the insertion and expression of foreign genes. Three foreign genes, including eGFP, iLOV3, and TEVp, were inserted into the intergenic junction between nsp1β and nsp2 and expressed by the respective recombinant PRRSVs (rPRRSV-SH01-eGFP, rPRRSV-SH01-iLOV3, and rPRRSV-SH01-TEVp) in vitro in mammalian cells. Analysis of the growth kinetics of the rescued recombinant PRRSVs showed no significant differences between the recombinant PRRSVs and their parental viruses. The inserted genes were consistently present in the viral genome during serial passage in vitro (for at least 20 passages). In addition, rPRRSV-SH01-eGFP can be used as a reporter virus for rapid detection of neutralizing antibodies against PRRSV through a fluorescent focus unit reduction-based assay. These data demonstrate that the junction between nsp1β and nsp2 is a new site that is suitable for the insertion and expression of foreign genes, providing a new option to express and deliver foreign genes using PRRSV-based vectors for different purposes, such as the development of multi-valent vaccines against PRRSV and other porcine viruses.

## 1. Introduction

Porcine reproductive and respiratory syndrome virus (PRRSV) is the causative agent of porcine reproductive and respiratory syndrome—an acute infectious disease characterized by reproductive failure in pregnant sows and respiratory disorders in growing and finishing pigs—and is considered a major pathogen affecting the global swine industry [1]. PRRSV, which belongs to the family *Arteriviridae* of the order *Nidoviridae*, along with equine arteritis virus, lactate dehydrogenase elevation virus, and hominid hemorrhagic fever virus [2], is an enveloped, positive single-stranded RNA virus. The genome of PRRSV is approximately 15 kilobases (kb) in length and consists of at least 10 open reading frames (ORFs) including ORF1a, ORF1b, ORF2a, ORF2b, ORF3-7, and ORF5a and two untranslated regions (UTRs) at the 5′- and 3′-ends of the genome [3].

ORF1a and ORF1b account for three-quarters of the PRRSV genome and encode large replicase polyproteins including polyprotein 1a (pp1a), pp1a-nsp2N, pp1a-nsp2TF, and pp1ab [4]. After translation from ORF1a, pp1a is proteolytically processed into 12 non-structural proteins (nsps) including nsp1α, nsp1β, nsp2, nsp2N, nsp2TF, nsp3-nsp6, nsp7α, nsp7β, and nsp8. The production of polyprotein 1ab (pp1ab) is accomplished by a ribosome-shifting mechanism at the ORF1a–ORF1b junction, which is proteolytically cleaved into four non-structural proteins (nsp9–12). The non-structural proteins encoded by ORF1a and ORF1b are the major components of the viral replication/transcriptase complex and are involved in transcription and replication of the viral genome [5]. ORF2a, ORF2b, ORF3-5, ORF5a, ORF6, and ORF7 encode the viral structural proteins GP2, E, GP3, GP4, GP5, and GP5a; the matrix protein (M); and the nucleocapsid protein (N); all of these are functionally responsible for constitution of the PRRSV virion and most of them have been associated with host immune response stimulation [6].

PRRSV has been considered as a promising viral vector to express and deliver foreign genes based on its full-length infectious cDNA clone, which was initially described using an RNA transfection strategy in 2000 [7]. The possible sites for the insertion of foreign genes include the non-essential regions of nsp2, the ORF7-coding region, and the intergenic junctions between ORF1b and ORF2a, ORF7 and 3′-UTR, and ORF4 and ORF5a [8,9]. Various foreign genes can be inserted depending on the intended purposes, including marker genes for the construction of reporter viruses or marked PRRSV vaccines [10,11,12], foreign antigens for the development of multi-valent vaccines against PRRSV and other porcine viruses [13,14], or immunomodulatory factors for enhanced vaccine efficacy against PRRSV infection [15,16]. For example, enhanced green fluorescent protein (eGFP) has been inserted into the non-essential region of nsp2 to construct a recombinant GFP-tagged PRRSV for analyses of virus replication, as well as to mark a vaccine [17]. Similarly, the coding sequence for Gaussia luciferase has been inserted into the intergenic junction between ORF7 and the 3′-UTR to generate a recombinant PRRSV for antiviral drug screening and luciferase-based neutralization assays [18]. The E2 gene of classical swine fever virus has been inserted the intergenic junction between ORF1b and ORF2 in a PRRSV vaccine strain for the development of multi-valent vaccines against PRRSV and classical swine fever virus [13]. The granulocyte-macrophage colony-stimulating factor (GM-CSF) gene and the synthesized TRS6 sequence have been inserted between ORF1b and ORF2 of a PRRSV vaccine strain to generate a recombinant vaccine that is capable of stimulating stronger immune responses and enhancing the efficacy of vaccines against PRRSV infection [15].

Although several insertion sites for the expression of foreign genes have been identified in the PRRSV genome [8], the genetic instability of some recombinant PRRSVs, characterized by partial or complete loss of the inserted gene(s) during serial passage in cultured cells, has occasionally ben described [9,19,20], which is partially attributable to the toleration capacity of the insertion sites, thus limiting the range of possible inserted genes and further applications. Therefore, identification of new insertion sites in the PRRSV genome is essential for using PRRSV as a vector for the expression and delivery of foreign genes. In this study, the junction between nsp1β and nsp2 was identified as a new site that is suitable for the insertion and expression of foreign genes.

## 2. Materials and Methods

### 2.1. Cell, Virus, and Serum Samples

Marc-145 and BHK-21 cells were cultured in Dulbecco’s Modified Eagle Medium (DMEM, Thermo Fisher Scientific, Waltham, MA, USA) supplemented with 10% fetal bovine serum (FBS, Thermo Fisher Scientific, Waltham, MA, USA) in a 37 °C incubator with 5% CO_2_. BHK-21 cells were used to rescue the virus by transfection with the full-length infectious cDNA, and Marc-145 cells were used for virus amplification and subsequent experiments [21,22]. The HP-PRRSV SH01 strain was isolated in our laboratory (GenBank accession number: PV424059). The pig serum samples used in the fluorescent focus unit reduction-based assay were collected on Day 35 after piglets were immunized with the rPRRSV-nsp4-mut10 strain as described previously [23].

### 2.2. Construction of a Full-Length Infectious cDNA Clone of the HP-PRRSV SH01 Strain

**Figure 1:**
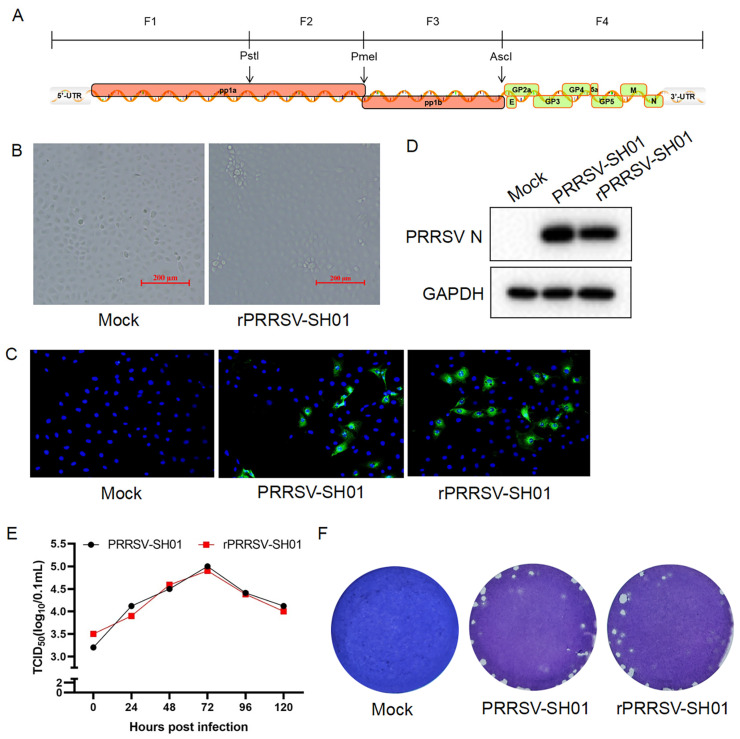
Construction of the recombinant virus rPRRSV-SH01. (**A**) Schematic representation of the four segments of the PRRSV-SH01 genome to be amplified and assembled for generation of a full-length infectious cDNA clone of pPRRSV-SH01. (**B**) Marc-145 cells were inoculated with the supernatants from BHK-21 cells transfected with the full-length cDNA clone of pPRRSV-SH01. The cytopathic effect (CPE) was visualized at 96 h post-infection under a light microscope. (**C**,**D**) Marc-145 cells were inoculated with the supernatants harvested from the CPE-presenting cells infected with rPRRSV-SH01. Expression of the PRRSV N protein was detected at 24 h post-infection via an immunofluorescence assay (**C**) with anti-N antibodies (green). Nuclei were stained with DAPI (blue). The expression of PRRSV N protein was further detected at 24 h post-infection via Western blotting with anti-N antibodies. GAPDH served as a loading control. Mock and PRRSV-SH01 served as the negative and positive controls, respectively. (**E**) Growth kinetics of rPRRSV-SH01 in Marc-145 cells. Marc-145 cells were infected with rPRRSV-SH01 or its parental PRRSV-SH01 strain at 0.1 MOI, and the supernatants were harvested at the indicated time intervals. Each virus titer was measured via a TCID_50_ assay. (**F**) Plaque assays. Monolayers of Marc-145 cells were infected with rPRRSV-SH01 or its parental PRRSV-SH01 strain. The plaques were stained with crystal violet at 72 h post-infection.

Construction of the full-length infectious cDNA clone was performed as described previously [24]. Briefly, viral RNA of HP-PRRSV SH01 was extracted with TRIzol^™^ (Thermo Fisher Scientific, Waltham, MA, USA) and reverse-transcribed into cDNA using StarScript III RT MasterMix (GENSTAR, Beijing, China). The genome of the HP-PRRSV SH01 strain was divided into four segments (F1–F4) approximately 3–5 kb in size at the unique restriction enzyme sites (*Pst* I, *Pme* I, and *Asc* I; Figure 1A) and amplified using primers (Table 1) designed on the basis of the genome covering the full length of the HP-PRRSV SH01 strain without the poly (A) tail using 2 × Phanta Max Master Mix (Vazyme, Nanjing, China). The PCR-amplified fragments were gel-purified with an E.Z.N.A^®^ Gel Extraction Kit (OMEGA Bio-tek, Norcross, GA, USA) and inserted into different intermediate vectors. Fragment 1 (F1) was inserted into the pCI vector digested with restriction enzymes (*Sac* I and *Not* I), and Fragment 2 (F2) was inserted into the pClone007 vector linearized by *Pst* I in order to construct the intermediate plasmid pCI-F1 and pClone007-F2, respectively. Fragment 3 (F3) was cyclized using the pClone007 Blunt Simple Vector Kit (TSINGKE, Beijing, China) directly in order to generate the intermediate plasmid pClone007-F3. Fragment 4 (F4) and a 337 bp fragment containing a polyadenosine (42A) and HDV ribozyme followed by an SV40 polyadenylation signal [25] amplified from the pOK-C34-EP plasmid were assembled into pUC19 digested by the restriction enzymes *Eco*R I and *Hin*d III to construct the intermediate plasmid pUC19-F4-42A. For assembly of the full-length infectious cDNA clone of pPRRSV-SH01, the F1 fragment digested from the intermediate plasmid pCI-F1 with the restriction enzymes *Nde* I and *Mre* I was inserted into the modified low-copy vector pACYC-177 to generate the plasmid pPRRSV-SH01-F1. The F2 fragment digested from the intermediate plasmid pClone007-F2 with the restriction enzymes *Pst* I and *Pme* I was ligated into the plasmid pPRRSV-SH01-F1 to construct the plasmid pPRRSV-SH01-F1-F2. The F3 fragment digested from the intermediate plasmid pClone007-F3 with the restriction enzymes *Pme* I and *Mre* I was introduced into the plasmid pPRRSV-SH01-F1-F2 to generate the plasmid pPRRSV-SH01-F1-F2-F3. The F4 fragment digested from the intermediate plasmid pUC19-F4-42A with the restriction enzymes *Asc* I and *Mre* I was inserted into the plasmid pPRRSV-SH01-F1-F2-F3 to obtain the plasmid pPRRSV-SH01 harboring the full-length cDNA of the HP-PRRSV SH01 strain.

### 2.3. Construction of Full-Length Infectious cDNA Clones of Recombinant PRRSV Expressing Foreign Genes Inserted Between nsp1β and nsp2

**Figure 2:**
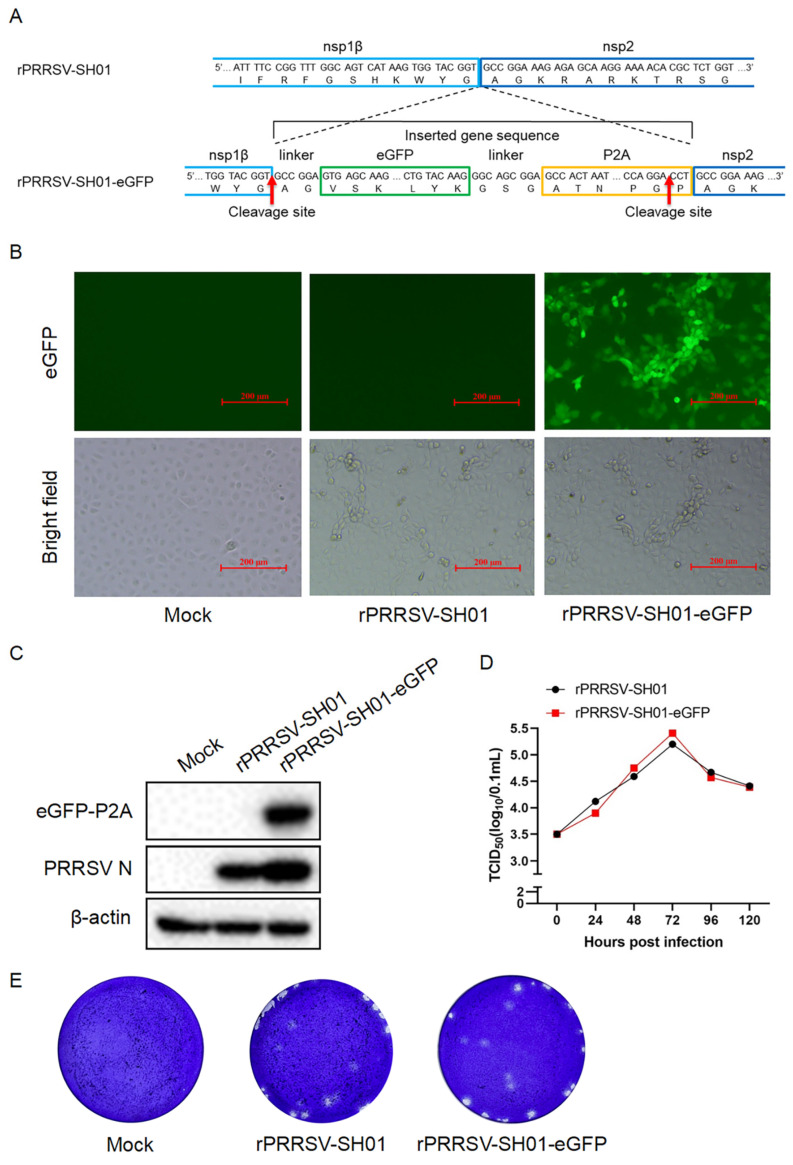
Construction of the recombinant virus rPRRSV-SH01-eGFP expressing eGFP. (**A**) Schematic representation of construction of rPRRSV-SH01-eGFP expressing eGFP inserted between nsp1β and nsp2. P2A indicates the porcine teschovirus-1 2A peptide. (**B**,**C**) Marc-145 cells were inoculated with the supernatants harvested from the transfected BHK-21 cells. The green fluorescence (eGFP) and CPE (brightfield) were visualized at 72 h post-infection under an inverted fluorescence microscope and a light microscope, respectively (**B**). The expression of eGFP-P2A and PRRSV N proteins was detected at 24 h post-infection via Western blotting with anti-eGFP and anti-N antibodies, respectively; β-actin served as a loading control (**C**). (**D**) Growth curve of rPRRSV-SH01-eGFP and its parental rPRRSV-SH01 strains. Marc-145 cells were infected with rPRRSV-SH01-eGFP and rPRRSV-SH01 strains at 0.1 MOI, and the supernatants were harvested at the indicated time intervals. Virus titers were measured via a TCID_50_ assay. (**E**) Plaque morphology of rPRRSV-SH01-eGFP and its parental rPRRSV-SH01 strain. Monolayers of Marc-145 cells were infected with rPRRSV-SH01-eGFP and its parental strain, respectively. The plaques were stained with crystal violet at 72 h post-infection.

Foreign genes, including eGFP, iLOV3, and TEVp, fused with the P2A gene via linker sequences were amplified through PCR using 2 × Phanta Max Master Mix (Figure 2A). The gene sequences encoding the C-terminus of nsp1β and the N-terminus of nsp2 were amplified, respectively, using the cDNA of HP-PRRSV SH01 as the template and subsequently assembled with the amplified eGFP-P2A gene via overlap extension PCR. The resulting fragment was inserted into the plasmid pCI-F1 (*Nar* I and *Eco*R I) to generate the plasmid pCI-F1-eGFP. For the construction of the intermediate plasmids pCI-F1-iLOV3 and pCI-F1-TEVp, the gene sequences encoding the C-terminus of nsp1β and the P2A followed by the N-terminus of nsp2 were amplified using the plasmid pCI-F1-eGFP as a template and subsequently assembled with the amplified iLOV3 or TEVp gene via overlap extension PCR. The resulting digested fragments (*Nar* I and *Eco*R I) were inserted into the plasmid pCI-F1 to generate the plasmids pCI-F1-iLOV3 and pCI-F1-TEVp. The sequences encoding target genes from the plasmid pCI-F1-target gene were inserted into the full-length infectious cDNA clone of pPRRSV-SH01-42A to construct the full-length infectious cDNA clones of recombinant PRRSVs expressing foreign genes inserted between nsp1β and nsp2 (i.e., pPRRSV-SH01-eGFP, pPRRSV-SH01-iLOV3, and pPRRSV-SH01-TEVp).

### 2.4. Rescue of Recombinant PRRSVs

Rescue of recombinant PRRSVs was performed as described previously [26]. Briefly, the full-length infectious cDNA clones of recombinant PRRSVs (1 μg per well) were transfected into BHK-21 cells at 80% confluence in a 24-well cell culture plate using TurboFect™ transfection reagent (Thermo Fisher Scientific, Waltham, MA, USA), according to the manufacturer’s instructions. The transfected BHK-21 cells were cultured in DMEM supplemented with 2% FBS for 24 h, following which, the supernatants were replaced with fresh DMEM supplemented with 2% FBS for an additional 24 h of culture. The harvested supernatants were inoculated into Marc-145 cells for amplification of the rescued recombinant virus. This process was repeated 4 times at 24 h intervals or until the appearance of the typical CPE of PRRSV infection in Marc-145 cells. If no CPE appeared, the supernatants harvested from Marc-145 cells were blindly passaged in Marc-145 cells 3 times for amplification of the rescued recombinant virus.

### 2.5. Plaque Assays

Plaque assays were performed as described previously [27] with minor modifications. Briefly, monolayers of Marc-145 cells grown in 6-well culture plates were incubated with serial dilutions of viral inocula at 37 °C for 2 h. The inocula were replaced with 1% LMP agarose (Thermo Fisher Scientific) in DMEM with 2% FBS, and the plates were incubated at 37 °C with 5% CO_2_ following agarose solidification at room temperature. The cells were fixed with 4% paraformaldehyde at 72 h post-inoculation and stained with 1% crystal violet. The plaque morphology was compared between viruses.

### 2.6. Immunofluorescence Assay, Western Blotting, and RT–PCR

Immunofluorescence assays and Western blotting were performed as described previously [27]. Antibodies specific to PRRSV N were used for detection of the expression of PRRSV N in the immunofluorescence assays and Western blots. Antibodies specific to eGFP and TEVp were used for detection of the expression of eGFP and TEVp proteins in Western blots. The foreign genes inserted between nsp1β and nsp2 were detected via RT–PCR amplification. Briefly, viral RNA extraction and reverse transcription were performed as described above. The sequences of foreign genes flanked with partial nsp1β and nsp2 were amplified via PCR using 2×Hieff^®^ PCR Master Mix with Dye (Yeasen, Shanghai, China) using specific primers (Table 1). The PCR cycling conditions were 95 °C for 5 min, then 35 cycles of 95 °C melting (30 s), 65 °C annealing (30 s), and 72 °C extension (1 kb per min) and, finally, 72 °C for 10 min.

### 2.7. Replication Kinetics of PRRSV and Viral Titration

The replication kinetics of PRRSV and viral titration were performed as described previously [23,24]. Briefly, Marc-145 cells were infected with PRRSV at 0.1 MOI and incubated at 37 °C for 120 h. The supernatants were sampled at the indicated time intervals and subjected to viral titration. For viral titration, Marc-145 cells grown in 96-well cell culture plates were infected with PRRSV diluted in 10-fold serial dilutions and monitored for CPE development. The virus titers in the supernatants were measured through a 50% tissue culture infectious dose (TCID_50_) assay, using the Reed–Muench method.

### 2.8. Fluorescence Focus Unit Reduction-Based Assay for Detection of Neutralizing Antibodies

The fluorescence focus unit reduction-based assay was performed as described previously [10]. Briefly, serum samples were inactivated at 56 °C for 30 min and subsequently serially two-fold diluted in DMEM starting at 1:4. Each dilution of the serum sample was mixed with an equal volume of PRRSV at a dose of 100 TCID_50_ and incubated at 37 °C for 1 h. Marc-145 cells prepared in 96-well plates were inoculated with the serum–virus mixture for 2 h at 37 °C. After adsorption, the inocula were discarded and the cells were maintained in DMEM containing 2% FBS at 37 °C in an atmosphere containing 5% CO_2_. The fluorescence signal of each well was read on a multi-detector microplate reader (SpectraMax M3; Molecular Devices, San Jose, CA, USA) at Ex/Em = 485/535 nm at 48 h post-inoculation. The neutralizing antibody titer was determined as the reciprocal of the highest serum dilution that reduced the number of fluorescent focus units by at least 90% relative to the virus control [10,28,29,30].

### 2.9. Statistical Analysis

All statistical analysis was performed by GraphPad Prism (version 8.0) software. Statistical significance was evaluated by a two-tailed unpaired Student’s *t* test; *p* < 0.05 was considered statistically significant.

## 3. Results

### 3.1. Construction and Recovery of the Recombinant Virus rPRRSV-SH01

The genome of the HP-PRRSV SH01 strain was divided into four segments of approximately 3–5 kb at unique restriction enzyme sites (*Pst* I, *Pme* I, and *Asc* I) and amplified via RT–PCR (Figure 1A). The resulting four segments were inserted sequentially into a low-copy plasmid using pACYC-177 as the backbone and subsequently assembled to generate a full-length infectious cDNA clone named pPRRSV-SH01. The accuracy and integrity of pPRRSV-SH01 were confirmed through Sanger sequencing. BHK-21 cells were transfected with pPRRSV-SH01, and the supernatants were harvested to infect Marc-145 cells for amplification of the recombinant virus. The typical cytopathic effect (CPE) of PRRSV infection appeared in the infected Marc-145 cells at 96 h post-infection (Figure 1B), and the supernatants were harvested when 80% CPE developed. The harvested supernatants were further inoculated into fresh Marc-145 cells to confirm the presence of rescued recombinant PRRSV (rPRRSV-SH01) via immunofluorescence assays (IFAs) and Western blotting. The expression of PRRSV N protein was detected in the rPRRSV-SH01-inoculated cells through both IFAs (Figure 1C) and Western blotting (Figure 1D) with antibodies specific to the PRRSV N protein, observing expression levels similar to those expressed in cells infected with its parental virus (PRRSV-SH01).

The growth kinetics of rPRRSV-SH01 were compared with its parental PRRSV-SH01 strain in Marc-145 cells, as described previously [24]. The multiple-step growth curve of rPRRSV-SH01 was similar to that of its parental strain (Figure 1E), and no significant difference was found between rPRRSV-SH01 and its parental strain. In addition, the sizes and shapes of plaques formed by rPRRSV-SH01 were also similar to those formed by its parental strain (Figure 1F). Taken together, these results demonstrate that the rPRRSV-SH01 was constructed and rescued with growth characteristics similar to those of its parental PRRSV-SH01 strain.

### 3.2. Construction and Recovery of the Recombinant Virus rPRRSV-SH01-eGFP Expressing eGFP Inserted Between nsp1β and nsp2

PRRSV nsp1β is autoproteolytically cleaved from the viral pp1a immediately after translation of pp1a [31] by its *cis*-active papain-like cysteine protease at the site (383G↓A384) between nsp1β and nsp2 [32], serving as a multi-functional protein interconnecting various stages of the PRRSV replication cycle. Therefore, the junction between nsp1β and nsp2 might be an additional candidate site for the insertion of foreign genes in the PRRSV genome. To test this hypothesis, eGFP fused with porcine teschovirus-1 2A peptide (P2A) linked by the amino acid sequence “GSG” (eGFP-P2A) was inserted into the intergenic junction between nsp1β and nsp2, based on the backbone of rPRRSV-SH01, in order to generate a full-length infectious cDNA clone of pPRRSV-SH01-eGFP (Figure 2A). A peptide linker with the amino acid sequence “AG” from the N-terminus of nsp2 was fused to the N-terminus of eGFP-P2A to ensure correct cleavage between the C-terminus of nsp1β and the N-terminus of eGFP-P2A.

P2A is a self-cleaving peptide (19 amino acids) that self-cleaves at the site between glycine (G) and praline (P) (G↓P) at its C-terminus (Figure 2A) [33] and is used to cleave eGFP-P2A from nsp2. The peptide linker of “GSG” between eGFP and P2A was used to increase the efficiency of P2A-mediated self-cleavage [34]. The eGFP-P2A is removed from nsp2 and the proline residue of P2A remains at the N-terminus of nsp2 after this P2A-mediated self-cleavage.

BHK-21 cells were transfected with the full-length infectious cDNA clones of pPRRSV-SH01-eGFP and pPRRSV-SH01. Subsequently, the supernatants were harvested to infect Marc-145 cells for virus amplification. The typical CPE of PRRSV infection was observed in both Marc-145 cells infected with rPRRSV-SH01-eGFP and its parental rPRRSV-SH01 virus (Figure 2B, brightfield panels). As expected, green fluorescence appeared only in Marc-145 cells infected with rPRRSV-SH01-eGFP and not in the cells infected with rPRRSV-SH01 (Figure 2B, eGFP panels). The supernatants harvested from the infected Marc-145 cells when 80% CPE was developed were further inoculated into fresh Marc-145 cells to confirm the presence of the rescued recombinant PRRSV (rPRRSV-SH01-eGFP). Analysis of protein expression via Western blotting revealed that the eGFP was detected in Marc-145 cells infected with rPRRSV-SH01-eGFP but not in those infected with rPRRSV-SH01, despite PRRSV N being detected in both (Figure 2C). These data suggest that rPRRSV-SH01-eGFP was successfully constructed.

The growth kinetics of rPRRSV-SH01-eGFP were compared with its parental rPRRSV-SH01 strain in Marc-145 cells. The multiple-step growth curve of rPRRSV-SH01-eGFP was similar to that of its parental strain (Figure 2D), and no significant difference was found between rPRRSV-SH01-eGFP and its parental strain. Analysis of plaque morphology indicated that the sizes and shapes of plaques formed by rPRRSV-SH01-eGFP were similar to those formed by its parental strain (Figure 2E). Overall, these results demonstrated that rPRRSV-SH01-eGFP was successfully constructed and rescued, presenting growth characteristics similar to its parental rPRRSV-SH01 strain, and that the junction between nsp1β and nsp2 can be considered as a novel site for the insertion of foreign genes.

### 3.3. Analysis of the Genetic Stability of rPRRSV-SH01-eGFP

**Figure 3:**
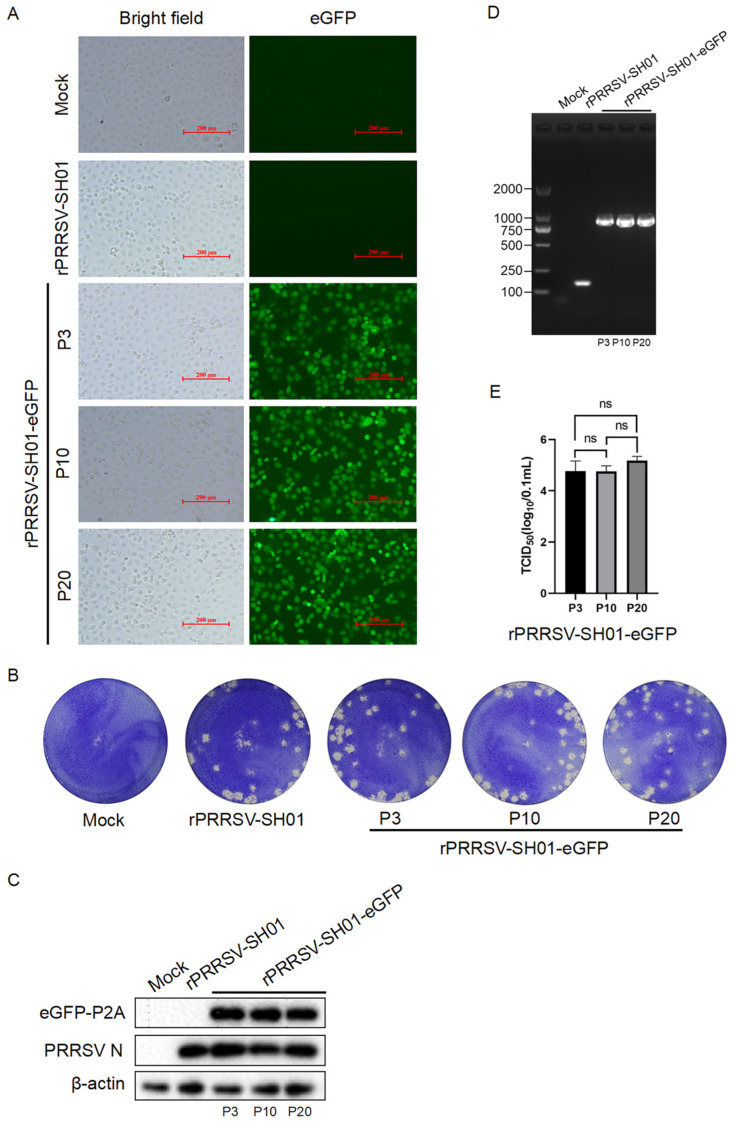
Genetic stability of rPRRSV-SH01-eGFP. rPRRSV-SH01-eGFP was serially passaged in Marc-145 cells and the 3rd (P3), 10th (P10), and 20th (P20) passages were selected to determine its genetic stability. (**A**) CPE (brightfield) and green fluorescence (eGFP) were visualized at 72 h post-infection under a light microscope and an inverted fluorescence microscope, respectively. (**B**) Plaque morphology of rPRRSV-SH01-eGFP of each of the selected passages. Monolayers of Marc-145 cells were infected with each of the selected passages of rPRRSV-SH01-eGFP. The plaques were stained with crystal violet at 72 h post-infection. (**C**) The expression of eGFP-P2A and PRRSV N proteins was detected at 24 h post-infection via Western blotting with anti-eGFP and anti-N antibodies, respectively; β-actin served as a loading control. (**D**) The sequence of eGFP-P2A flanked with partial nsp1β and nsp2 was amplified via RT–PCR. Mock and rPRRSV-SH01 served as controls. (**E**) Infectious titers in each of the selected passages were determined using a TCID_50_ assay. ns, no significant difference determined by Student’s *t* test.

rPRRSV-SH01-eGFP was serially passaged in Marc-145 cells 20 times, and the 3rd (P3), 10th (P10), and 20th (P20) passages were selected to determine its genetic stability. CPE and green fluorescence were observed in Marc-145 cells infected with each of the selected passages of rPRRSV-SH01-eGFP (Figure 3A). The shapes and sizes of plaques formed by rPRRSV-SH01 at P3, P10, and P20 were also similar to each other (Figure 3B). Analysis of the expression of eGFP and PRRSV N proteins via Western blotting indicated consistent expression levels of both proteins in each of the selected passages, and no remarkable differences were observed between the passages (Figure 3C).

In addition, the sequence of eGFP-P2A flanked with partial nsp1β and nsp2 was amplified from each of the selected passages using RT–PCR with specific primers (Table 1). As shown in Figure 3D, the sizes of the viral genomic regions amplified from each of the selected passages were identical and remarkably larger than those amplified from its parental rRPRRSV-SH01 strain.

The viral genomic regions amplified from each of the selected passages were further subjected to Sanger sequencing. The presence of the inserted sequence of eGPF-P2A was confirmed in each of the selected passages, and no additional nucleotide mutations or deletions emerged, indicating the consistent presence of eGFP-P2A in the viral genome during the serial passages.

The infectious titers in each of the selected passages were titrated via a TCID_50_ assay, which were approximately 10^5^ TCID_50_/0.1 mL, and no significant difference was observed among the selected passages (Figure 3E). Taken together, these data demonstrate that the eGFP-P2A gene inserted between nsp1β and nsp2 of the PRRSV genome remained genetically stable during serial passage in vitro (for at least 20 passages).

### 3.4. Application of rPRRSV-SH01-eGFP as a Reporter Virus for Detection of Immune Response Against PRRSV in Vitro

**Figure 4:**
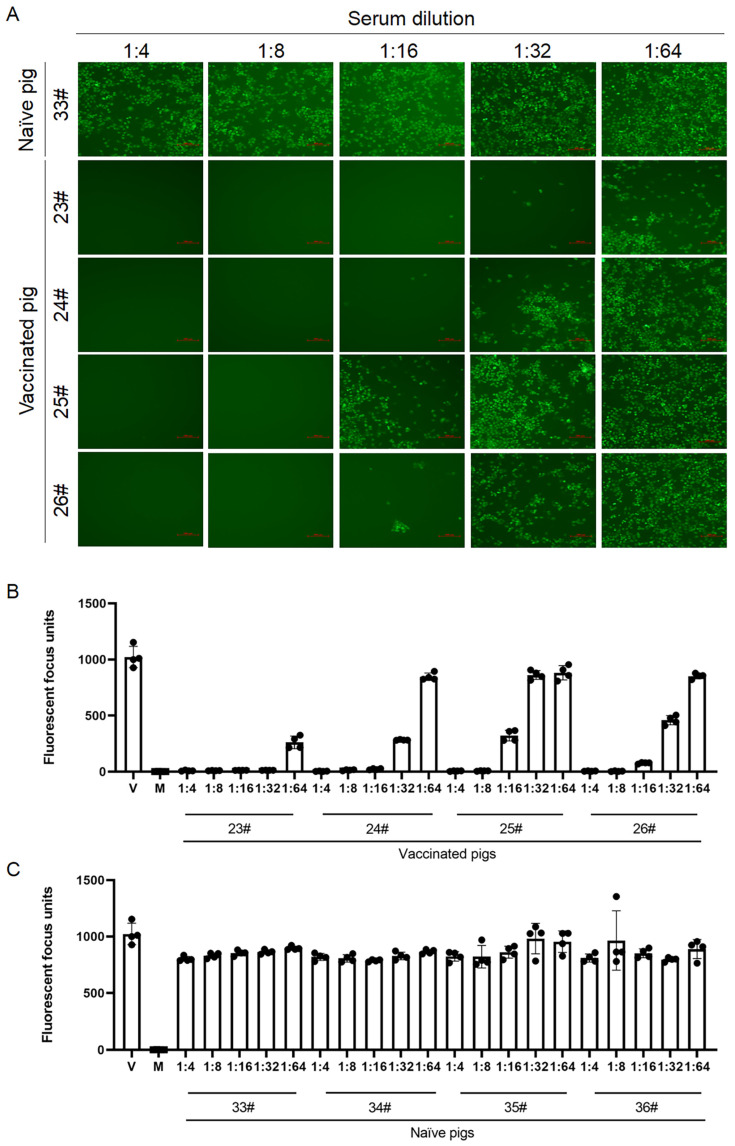
Fluorescent focus unit reduction-based assay for detection of neutralizing antibodies against PRRSV. The fluorescent focus unit reduction-based assay was performed using rPRRSV-SH01-eGFP at a dose of 100 TCID_50_ and serum samples diluted from 1:4 to 1:64. The fluorescent foci were captured using a multi-detector microplate reader at 48 h post-inoculation. (**A**) Green fluorescence of the tested samples was visualized under an inverted fluorescence microscope. Scale bar: 200 μm. (**B**) Fluorescent focus units of the serum samples from vaccinated pigs. (**C**) Fluorescent focus units of the serum samples from naïve pigs. Data are expressed as the mean ± standard deviation (SD). V, cells infected with rPRRSV-SH01-eGFP alone as a virus control; M, mock-infected cells as a negative control.

To take advantage of rPRRSV-SH01-eGFP expressing eGFP, it could be used as a reporter virus for detection of the immune response against PRRSV and anti-PRRSV component screening, similar to applications described previously for other recombinant PRRSV-expressing foreign genes [10,35]. Therefore, the rPRRSV-SH01-eGFP was employed to measure the neutralizing antibodies against PRRSV. To this end, a fluorescent focus unit reduction-based assay [28,30] was performed using rPRRSV-SH01-eGFP and serum samples from pigs immunized with a PRRSV vaccine. Reductions in the green fluorescent foci were visualized in the serum samples from vaccinated pigs with a variety of reduction rates, but not in the serum samples from naïve pigs at 48 h post-inoculation (Figure 4A). The fluorescent foci of the tested serum samples were captured using a multi-detector microplate reader and calculated as fluorescent focus units. The fluorescent focus units were remarkably reduced in the serum samples from vaccinated pigs, as compared with those in cells inoculated with rPRRSV-SH01-eGFP alone (Figure 4B). The fluorescent focus units were slightly reduced in the serum samples from naïve pigs, as compared with those cells inoculated with rPRRSV-SH01-eGFP alone (Figure 4C), which is likely attributable to the non-specific inhibition by unknown factors in pig sera.

On the basis of the fluorescent focus units, the neutralizing antibody titers of the tested serum samples were calculated as the reciprocal of the highest dilution that reduced the fluorescent focus units by at least 90% relative to the virus control [10,30]. As shown in Table 2, the neutralizing antibody titers of serum samples 23#, 24#, 25#, and 26# from vaccinated pigs were 1:32, 1:16, 1:8, and 1:16, respectively, and no neutralizing antibodies were observed in the serum samples obtained from naïve pigs. Overall, these data suggest that rPRRSV-SH01-eGFP could be used in fluorescent focus unit reduction-based assays for the detection of neutralizing antibodies against PRRSV.

### 3.5. Construction and Recovery of the Recombinant Virus rPRRSV Expressing iLOV3 or TEVp Inserted Between nsp1β and nsp2

**Figure 5:**
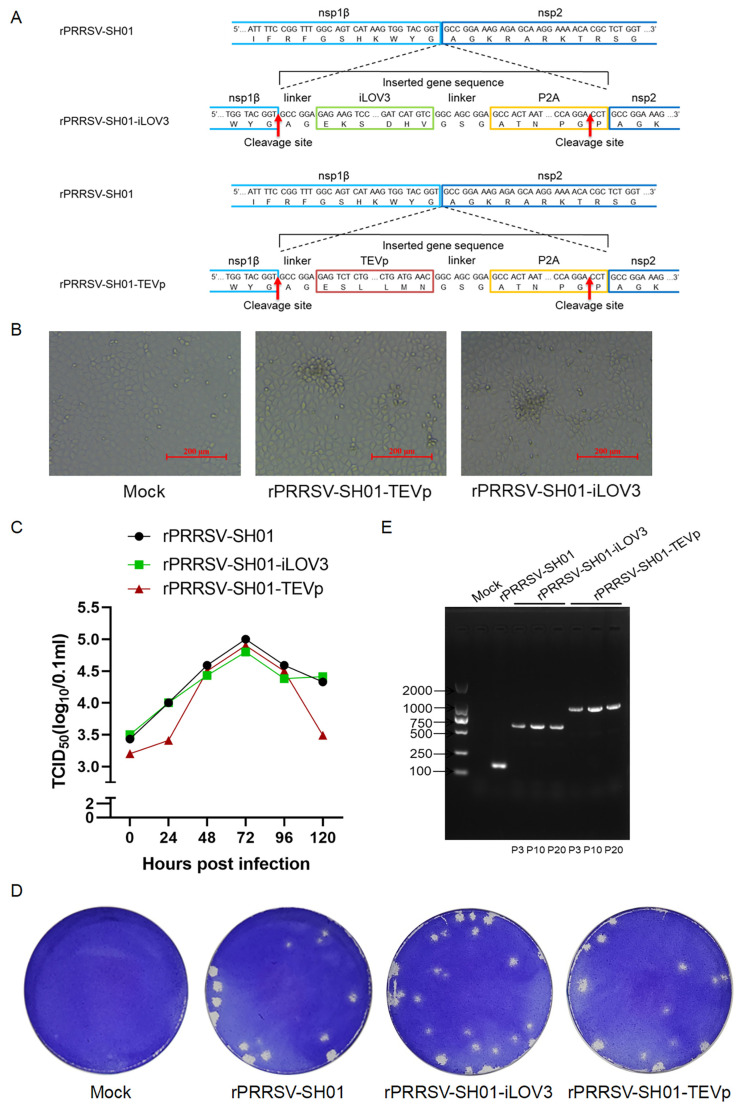
Construction and recovery of rPRRSV-SH01-iLOV3 and rPRRSV-SH01-TEVp. (**A**) Schematic representation of the construction of rPRRSV-SH01-iLOV3 (above) and rPRRSV-SH01-TEVp (below) expressing iLOV3 or TEVp, respectively, inserted between nsp1β and nsp2. P2A indicates the porcine teschovirus-1 2A peptide. (**B**) Marc-145 cells were inoculated with the supernatants harvested from the transfected BHK-21 cells. The CPEs were visualized at 72 h post-infection under a light microscope. (**C**) Growth curves of rPRRSV-SH01-iLOV3, rPRRSV-SH01-TEVp, and their parental rPRRSV-SH01 strain. Marc-145 cells were infected with rPRRSV-SH01-iLOV3, rPRRSV-SH01-TEVp, or the rPRRSV-SH01 strain at 0.1 MOI, and the supernatants were harvested at the indicated time intervals. Virus titers were measured via a TCID_50_ assay. (**D**) Plaque morphology of rPRRSV-SH01-iLOV3, rPRRSV-SH01-TEVp, and the parental rPRRSV-SH01 strain. Monolayers of Marc-145 cells were infected with rPRRSV-SH01-iLOV3, rPRRSV-SH01-TEVp, or the parental strain, respectively. The plaques were stained with crystal violet at 72 h post-infection. (**E**) Sequences of iLOV3-P2A or TEVp-P2A flanked with partial nsp1β and nsp2 were amplified via RT–PCR. Mock and rPRRSV-SH01 served as controls.

After confirming that eGFP inserted between nsp1β and nsp2 was stably expressed in rPRRSV-SH01-eGFP, the junction between nsp1β and nsp2 was further tested with respect to the insertion of additional foreign genes for stable expression. To this end, iLOV3 (a green fluorescent protein with a smaller size [36]) and TEVp (a cysteine protease from the tobacco etch virus with high efficiency and stringent substrate specificity [37]) were inserted into the intergenic junction between nsp1β and nsp2 based on the backbone of rPRRSV-SH01, using a strategy identical to that for the insertion of eGFP (Figure 5A). In this way, full-length infectious cDNA clones of pPRRSV-SH01-iLOV3 and pPRRSV-SH01-TEVp were constructed. The constructed full-length infectious cDNA clones of pPRRSV-SH01-iLOV3 and pPRRSV-SH01-TEVp were transfected into BHK-21 cells to rescue the recombinant viruses rPRRSV-SH01-iLOV3 and rPRRSV-SH01-TEVp, respectively. Subsequently, the rescued recombinant viruses were amplified in Marc-145 cells (Figure 5B).

Comparison of the growth kinetics of rPRRSV-SH01-iLOV3 with its parental rPRRSV-SH01 strain in Marc-145 cells showed that the multiple-step growth curve of rPRRSV-SH01-iLOV3 was similar to that of its parental strain (Figure 5C), and no significant difference was observed between the viruses. Meanwhile, the titers of rPRRSV-SH01-TEVp were similar to those of its parental rPRRSV-SH01 strain at 48 h, 72 h, and 96 h but lower than those at 24 h and 120 h post-infection (Figure 5C). And no significant differences were found in the shapes and sizes of plaques formed by rPRRSV-SH01-iLOV3, rPRRSV-SH01-TEVp, and the parental strain rPRRSV-SH01 (Figure 5D).

rPRRSV-SH01-iLOV3 and rPRRSV-SH01-TEVp were serially passaged in Marc-145 cells 20 times, and the third (P3), 10th (P10), and 20th (P20) passages were selected to determine their genetic stabilities. The sequence of the inserted iLOV3-P2A and TEVp-P2A flanked with partial nsp1β and nsp2 was amplified from each of the selected passages using RT–PCR with specific primers (Table 1). The sizes of the viral genomic regions amplified from each of the selected passages were identical (Figure 5E), and these viral genomic regions were further subjected to Sanger sequencing. The presence of the inserted sequence of iLOV3-P2A or TEVp-P2A was confirmed in each of the selected passages, and no additional nucleotide mutations or deletions emerged, indicating the consistent presence of iLOV3-P2A and TEVp-P2A in the viral genome during the serial passages. In addition, green fluorescence was observed in Marc-145 cells infected with each of the selected passages of rPRRSV-SH01-iLOV3 (Figure S1A). Analysis of the expression of TEVp and PRRSV N proteins via Western blotting indicated similar expression levels of both proteins in each of the selected passages, and no remarkable differences were observed between the passages (Figure S1B). Overall, these data further confirmed that the junction between nsp1β and nsp2 is a suitable site for the insertion of foreign genes.

## 4. Discussion

PRRSV is considered a promising viral vector for the expression and delivery of foreign genes for the development of a new generation of multi-valent vaccines against PRRSV and other porcine viruses, as well as for analyses of the immune response against PRRSV and anti-PRRSV component screening [8,10,13,14,18,35,38]. In this context, the identification of new insertion sites in the PRRSV genome is essential for using PRRSV as a vector for the expression and delivery of foreign genes. In this study, we found that the junction between nsp1β and nsp2 is a new site that is suitable for the insertion and expression of foreign genes, providing a new option for the expression and delivery of foreign genes using a PRRSV-based vector.

The junction site between nsp1β and nsp2, selected for the insertion of foreign genes in this study, is autoproteolytically cleaved by PRRSV *cis*-active papain-like cysteine protease immediately after translation of viral pp1a from the genomic mRNA (ORF1a) [31,32]. This proteolysis is independent of host proteases or other viral proteases (e.g., nsp4). In addition, the exact cleavage site (383G↓A384) between nsp1β and nsp2 has been identified previously [32]. The insertion of foreign genes into this site might not impair the normal processing of viral proteins and the biological functions of nsp1β and nsp2. Furthermore, the foreign genes were inserted into the intergenic site between nsp1β and nsp2 located in PRRSV ORF1a, which is translated at the beginning of viral replication without additional transcription units of PRRSV and could be expressed at the early stage of viral infection to achieve biological functions of interest. Therefore, we selected the junction site between nsp1β and nsp2 for the insertion and expression of foreign genes.

To ensure correct cleavage between the C-terminus of nsp1β and the inserted foreign proteins, a linker with the amino acid sequence “AG” from the N-terminus of nsp2 was fused to the N-terminus of foreign proteins. To cleave the inserted foreign proteins from the N-terminus of nsp2, the self-cleaving peptide P2A from porcine teschovirus-1 [33] was inserted between the foreign proteins and the N-terminus of nsp2. Ideally, the foreign proteins could be released as individual proteins from the viral protein after being proteolytically proceed by both PRRSV *cis*-active papain-like cysteine protease and P2A, and could be used for different purposes, such as the development of multi-valent vaccines against PRRSV and other porcine viruses.

In the present study, three foreign genes—eGFP, iLOV3, and TEVp—were inserted into the intergenic junction between nsp1β and nsp2 and expressed by the respective recombinant PRRSVs (rPRRSV-SH01-eGFP, rPRRSV-SH01-iLOV3, and rPRRSV-SH01-TEVp). We noted that a proline residue of P2A remained at the N-terminus of nsp2 after P2A-mediated self-cleavage. However, analysis of the growth kinetics of the rescued recombinant PRRSVs showed no significant differences between the recombinant PRRSVs and their parental viruses. In addition, the inserted genes were consistently present in the viral genome during serial passage in vitro. These data reveal that the insertion of foreign genes into the intergenic junction between nsp1β and nsp2 and the remaining proline residue of P2A at the N-terminus of nsp2 had no remarkable effects on the replication of PRRSV, suggesting that the junction between nsp1β and nsp2 is a novel site that is suitable for the insertion and expression of foreign genes.

As a viral vector, a number of sites in the PRRSV genome have been described previously for the insertion and expression of foreign genes. For example, the highly diverse and variable region of nsp2, which can tolerate some amino acid deletions, insertions, and mutations, has been described as an ideal site for the insertion of foreign genes [11,17,19,39]. In addition, the sites between ORF1b and ORF2a, ORF7 and 3′-UTR, and ORF4 and ORF5a [14,38,40] have also been shown to be suitable for the insertion and expression of different foreign genes. Despite these previous efforts, PRRSV-based vectors still face problems related to genetic instability, with some recombinant PRRSVs having been shown to lose (either partially or completely) the inserted gene(s) during serial passage in cultured cells [9,19,20]. This genetic instability is likely due to a number of factors, such as the toleration capacity of the inserted genetic material in the PRRSV genome, the size and genomic position of the inserted gene fragments, and the biological activities of the inserted gene products [9,19,20,22]. For example, the HA tag has been introduced into the N-terminus of PRRSV N protein using the F2A peptide in a previous study, indicating that foreign genes can be inserted into PRRSV ORF7 [7]. However, we failed to rescue a recombinant PRRSV with a foreign protein fused to the N-terminus of the PRRSV N protein using the P2A peptide. We also attempted to insert TEVp into the site between ORF1b and ORF2a using a unique transcription unit, but mutation and deletion were observed in the recombinant virus. In the present study, the junction between nsp1β and nsp2 was identified as a new site that is suitable for the insertion of three foreign genes (eGFP, iLOV3, and TEVp); however, more genes with different sizes and biological activities should be tested for determination of the potentials and limitations of this new site regarding the insertion and expression of foreign genes.

Recombinant PRRSVs expressing foreign genes, such as eGFP [35] and the Gaussia luciferase [18], have been used as reporter viruses in different applications, such as the determination of neutralizing antibodies against PRRSV. For measurement of neutralizing antibodies against PRRSV, the traditional virus neutralization test based on CPE has been used for a long time. This method is time-consuming, as it requires waiting for the appearance of CPE. The fluorescent focus neutralization assay has been additionally developed to measure neutralizing antibodies, which also requires a time-consuming process of an indirect immunofluorescent assay and manual foci counting requiring specialized technical expertise [28,29,30].

A luciferase-expressing reporter PRRSV has been constructed and used for the measurement of neutralizing antibodies against PRRSV through measurement of luciferase activity [18,38]. Although it can perform high-throughput analysis, this method requires a commercial detection kit for the measurement of luciferase activity. Similar to the present study, a Clover/eGFP-tagged PRRSV has been constructed for detection of neutralizing antibodies against PRRSV using a fluorescence microscope or a fluorescence reader [10,35]. In the present study, rPRRSV-SH01-eGFP was employed to measure the neutralizing antibodies against PRRSV through a fluorescent focus unit reduction-based assay [28,30]. The neutralization titers were calculated on the basis of the reduction in the number of fluorescent focus units [10,28,30]. The fluorescent focus units, which were automatically captured and calculated using a multi-detector microplate reader, were remarkably reduced in the serum samples from PRRSV-vaccinated pigs, as compared with those from naïve pigs, suggesting that rPRRSV-SH01-eGFP could be used as a reporter virus for rapid detection of neutralizing antibodies against PRRSV, providing an alternative method for the measurement of neutralizing antibodies against PRRSV. In addition to the measurement of neutralizing antibodies, the rPRRSV-SH01-eGFP could be used in different applications, such as antiviral drug screening and tracing of PRRSV replication in cells, as well as the determination of PRRSV–host interactions.

## 5. Conclusions

In conclusion, the junction site between nsp1β and nsp2 in the PRRSV genome was tested regarding its capacity for the insertion and expression of foreign genes. Three foreign genes—eGFP, iLOV3, and TEVp—were inserted into the intergenic junction between nsp1β and nsp2 and expressed by the respected recombinant PRRSVs (i.e., rPRRSV-SH01-eGFP, rPRRSV-SH01-iLOV3, and rPRRSV-SH01-TEVp). Analysis of the growth kinetics of the rescued recombinant PRRSVs showed no significant differences between the recombinant PRRSVs and their parental viruses. The inserted genes were consistently present in the viral genome during serial passage in vitro (for at least 20 passages). In addition, rPRRSV-SH01-eGFP could be used as a reporter virus for rapid detection of neutralizing antibodies against PRRSV through a fluorescent focus unit reduction-based assay. These data demonstrate that the junction between nsp1β and nsp2 is a new site that is suitable for the insertion and expression of foreign genes, providing a new option to express and deliver foreign genes using PRRSV-based vectors for different purposes, such as detection of neutralizing antibodies against PRRSV and development of multi-valent vaccines against PRRSV and other porcine viruses.

## Figures and Tables

**Table 1:** The primer pairs used in this study.

Name	Sequence (5′ to 3′)	Usage
PRRSV *Sac* I	ggtctatataagca*gagctc*gtttagtgaaccgt**atgacgtataggtgttgg**	Construction of the intermediate plasmid pCI-F1
PRRSV *Pst* I	ctgctcgaagcggc*cgccggcg*gagctttg*tttaaa*cccaa***ctgcag*ttacataaacac**	
PRRSV *Pst* I nsp3	atcgtcagtattga***ctgcag*tggggt**	Construction of the intermediate plasmid pClone007-F2
Infnsp8	gtgtctgaggctcgc**gctagca*gtttaaac*ac**	
1b *Pme* I	***gtttaaac*** **tgctagccg**	Construction of the intermediate plasmid pClone007-F3
1b *Asc* I	*cgccggcg*gaattcgatatcgaattca***ggcgcgcc*cgaaac**	
PRRSV *Asc* I	tgattacgccaagc**t*ggcgcgcc*ag**	Construction of the intermediate plasmid pUC19-F4-42A
PRRSV3UTR	**aattacggccgcatg**	
42AF	**catgcggccgtaattaaaaaaaaaaaaaaaaaaaaaaaaaaaaaaaaaaaaaaaaaa**gg	
InPRRSV SVR	gacggccagtgaatt*cgccggcg*ttaacttgtttattgcagc	
seq*Nar* I nsp2stu	**cccaaacctgaggac** **ccttactctttcaggaaggg**	Construction of the intermediate plasmid pCI-F1-target gene
1br 1bGFP P2Ar P2Ansp2	gcct**gcaccgtacc** **ggtacggtg**caggcgtgagcaagggc aggtcctgggttttcctccacgtctc gtggaggaaaacccaggacc**tgccggaaagagag**	Construction of the intermediate plasmid pCI-F1-eGFP
P2Af	ggcagcggagcc	Construction of the intermediate plasmids pCI-F1-iLOV3 and pCI-F1-TEVp
1biLOV3	**ggtacggtg**caggcgagaagtcctttgtg	Construction of the intermediate plasmid pCI-F1-iLOV3
iLOV3P2A	gtggctccgctgccgacatgatcggagccg	
1bTEVp	**ggtacggtg**caggcgagtctctgtttaagg	Construction of the intermediate plasmid pCI-F1-TEVp
TEVpP2A	gtggctccgctgccgttcatcagctgggtg	
nsp1bPLAG	**acgtcaccactggctgg**	Detection of inserted target genes
nsp2ATKH	**gcttcgtggcctgcc**	

Italicized letters represent the restriction enzyme sites. Bold letters depict the viral genome sequences.

**Table 2:** Percentage of reduction in fluorescent focus units.

	Percentage of Reduction in Fluorescent Focus Units (%)					
	Serum Sample	1:4	1:8	1:16	1:32	1:64
Vaccinated pigs	23#	99.27 ± 0.46	99.18 ± 0.16	98.95 ± 0.05	98.90 ± 0.08	74.54 ± 5.49
	24#	99.73 ± 0.22	98.76 ± 0.48	97.92 ± 0.56	72.44 ± 0.32	17.25 ± 3.22
	25#	99.58 ± 0.25	99.46 ± 0.21	68.66 ± 4.70	15.68 ± 3.88	13.74 ± 6.21
	26#	99.61 ± 0.17	99.71 ± 0.29	92.63 ± 0.61	55.20 ± 4.03	16.64 ± 2.34
Naïve pigs	33#	21.55 ± 2.14	18.81 ± 2.34	16.51 ± 2.35	15.61 ± 1.80	12.29 ± 1.68
	34#	19.89 ± 3.08	21.16 ± 3.25	23.00 ± 0.90	19.02 ± 3.29	15.34 ± 1.82
	35#	19.43 ± 4.15	19.67 ± 9.76	15.74 ± 5.20	4.02 ± 13.21	6.66 ± 9.32
	36#	20.82 ± 3.56	5.63 ± 25.72	16.79 ± 3.86	22.17 ± 1.77	13.12 ± 8.28

Dilutions reducing the fluorescent focus units by ≥90% relative to the virus control are highlighted in red.

## Data Availability

The virus genomic sequence used in the present work was deposited in the GenBank database of the National Center for Biotechnology Information (NCBI) under accession numbers (PV424059). Further data that support the findings of this study are available from the corresponding author upon request.

## Supplementary Materials

The following supporting information can be downloaded at: https://www.mdpi.com/article/10.3390/v17050656/s1. Figure S1: Genetic stability of rPRRSV-SH01-iLOV3 and rPRRSV-SH01-TEVp.
