# Supplementary material for: The Junction Between nsp1β and nsp2 in the Porcine Reproductive and Respiratory Syndrome Virus Genome Is a New Site for the Insertion and Expression of Foreign Genes
Source: Viruses. 2025 Apr 30;17(5):656. doi: 10.3390/v17050656 (PMC12115618; doi:10.3390/v17050656)
Supplement: Supplementary file 1 [file viruses-17-00656-s001.zip › viruses-3544457-supplementary.pptx]

## Slide 1
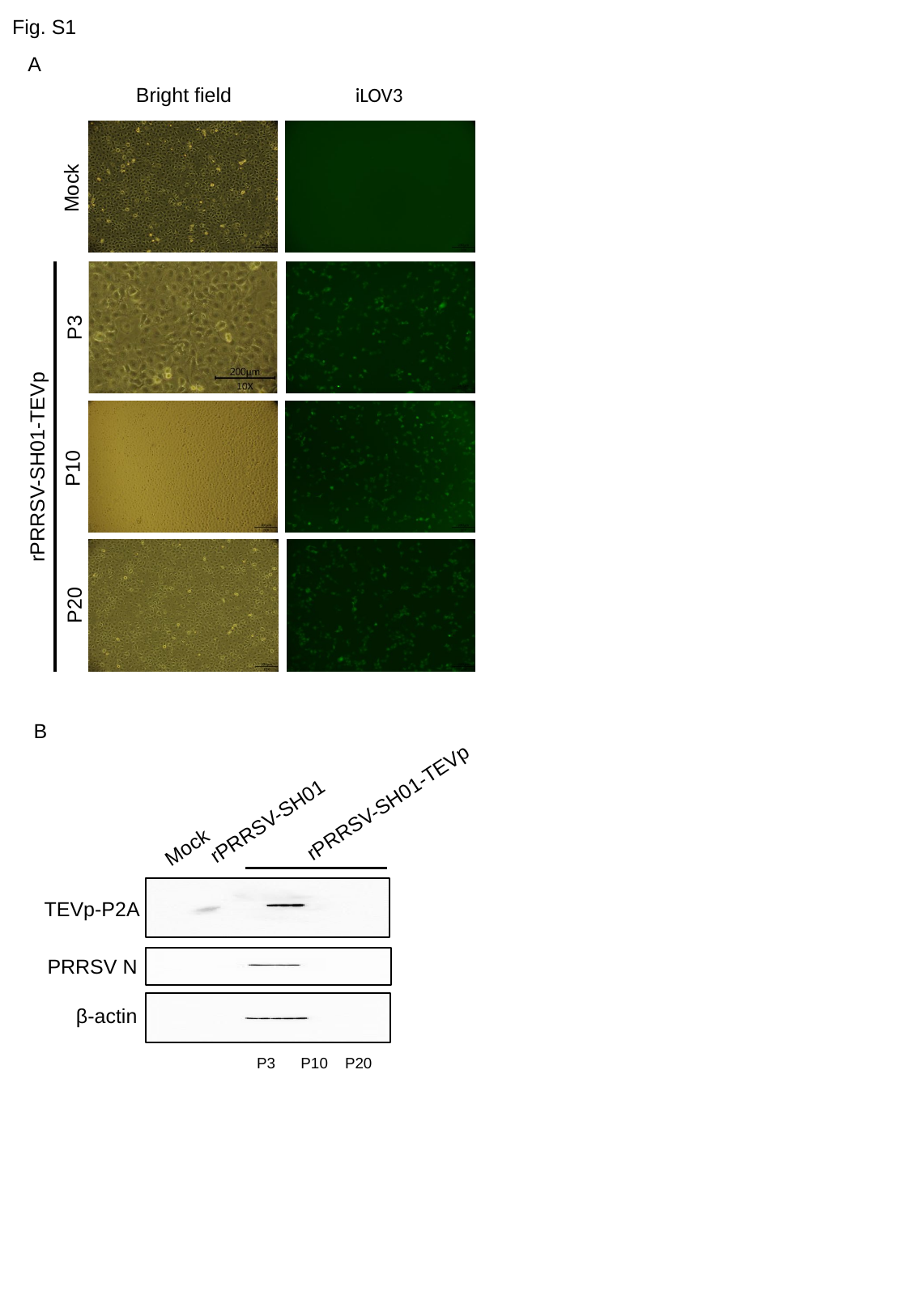

Fig. S1
iLOV3
Bright field
A
Mock
P3
P10
rPRRSV-SH01-TEVp
P20
B
rPRRSV-SH01-TEVp
rPRRSV-SH01
Mock
P3 P10 P20
TEVp-P2A
PRRSV N
β-actin
